# Role of Peripheral Inflammatory Markers in Postoperative Cognitive Dysfunction (POCD): A Meta-Analysis

**DOI:** 10.1371/journal.pone.0079624

**Published:** 2013-11-13

**Authors:** Linying Peng, Liwei Xu, Wen Ouyang

**Affiliations:** 1 Department of Anesthesiology, The Third Xiangya Hospital of Central South University, Changsha, China; 2 Department of Gastrointestinal Surgery, The First Xiangya Hospital of Central South University, Changsha, China; Chiba University Center for Forensic Mental Health, Japan

## Abstract

**Background:**

Postoperative cognitive dysfunction (POCD) is common following cardiac and non-cardiac surgery, but the pathogenic mechanisms remain unknown. Many studies suggest that an inflammatory response is a key contributor to POCD. The current meta-analysis shows that the levels of peripheral inflammatory markers are associated with POCD.

**Methods:**

An online search was performed to identify peer-reviewed studies without language restriction that measured peripheral inflammatory markers of patients with and without POCD, using PubMed, ScienceDirect, SinoMed and the National Knowledge Infrastructure database. Extracted data were analyzed with STATA (version 12).The standardized mean difference (SMD) and the 95% confidence interval (95%CI) were calculated for each outcome using a random effect model. Tests of heterogeneity assessment of bias, and meta-regression were performed in the meta-analysis.

**Results:**

A total of 13 studies that measured the concentrations of peripheral inflammatory markers were included. The current meta-analysis found significantly higher concentrations of S-100β(SMD[95%CI]) (1.377 [0.423, 2.331], p-value < 0.001, N [POCD/non-POCD] =178/391, 7 studies), and interleukin(IL)-6 (SMD[95%CI]) (1.614 [0.603,2.624], p-value < 0.001, N[POCD/non-POCD] = 91/99, 5 studies), but not of neuron specific enolase, interleukin-1β, or tumor necrosis factor-α , in POCD compared with patients without POCD. In meta-regression analyses, a significant positive association was found between the SMD and the preoperative interleukin-6 peripheral blood concentration in patients with POCD (Coef.= 0.0587, p-value=0.038, 5 studies).

**Conclusions:**

This study shows that POCD is indeed correlated with the concentrations of peripheral inflammatory markers, particularly interleukin-6 and S-100β.

## Introduction

Postoperative cognitive dysfunction (POCD) usually manifests as an alteration in orientation, memory, thinking, attention, insight or other aspects of central nervous function. Initially, it was thought to be associated with cardiac surgery. However, later studies showed that it has been associated with non-cardiac surgery and even with non-invasive procedures such as coronary angiography [1]. POCD can last for a few days to a few years. It decreases the patient’s quality of life and increases the cost of hospitalization and out-of-hospital care. It also increases surgical morbidity and mortality [2,3]. In patients over the age of 60, POCD was observed in 25.8% of patients at 1 week post-surgery and in 9.9% of patients at 3 months post-surgery [4].

Thus, POCD is an important concern for the anesthesiologist. 

Considerable evidence suggests that an inflammatory response may be involved in the occurrence of POCD [5,6]. In the clinic, anesthesiologists regularly study the correlation between POCD and factors such as S-100β protein(S-100β), neuron specific enolase(NSE), interleukin-1β(IL-1β), interleukin-6(IL-6), interleukin-8(IL-8), interleukin-10(IL-10), tumor necrosis factor(TNF)-α, and C-reactive protein(CRP). However, direct evidence showing a relationship between POCD and inflammatory markers is lamentably absent from the literature. Furthermore, opposing results are observed at same experimental conditions and experimental designs. Therefore, we conducted a meta-analysis to pool and analyze the data and to determine the relationship between POCD and specific inflammatory markers. 

## Materials and Methods

### Data Sources and Search Strategy

All analyses were performed according to PRISMA guidelines [7] and the Cochrane handbook for systematic reviews of interventions. PRISMA guidelines focus on randomized trials, but the PRISMA statement specifies that “PRISMA can also be used as a basis for reporting systematic reviews of other types of research”. Literature was searched using PubMed, ScienceDirect, SinoMed and the National Knowledge Infrastructure database. Searches were performed using the key words POCD and S-100β, NSE, IL-1β, IL-6, IL-8, TNF-α up to April 2013. All articles selected were included only human studies. The reference lists of relevant studies were searched for additional reports. No standardized review protocol has been published.

### Study Selection

Original studies measuring inflammatory marker concentrations in living subjects with POCD were included. Inclusion criteria were as follows: 1.) case-control study including non-POCD subjects as controls; 2.) human subjects; 3.) explicit diagnostic criteria such as the Confusion Assessment Method [8], the Digit-Symbol-Substitution Test of the Wechsler Adult Intelligence Scale (as a measure of attention) [2,9], and a diagnosis based on DSM-III criteria, examined using the Mini-mental State Examination [10,11] and so on.

Studies were excluded if: 1.) the article was a case report; 2.) the trials measured inflammatory marker concentrations from peripheral blood cells following additional stimulation; 3.) the trials did not group the results according to POCD status; 4.) the date were in an unavailable format.

### Data Extraction

Three independent raters examined each retrieved article. The results were compared between raters and any disagreements regarding inclusion were settled by consensus. The methods and results sections of each relevant article were analyzed, and cytokine concentration metrics for POCD and non-POCD groups were extracted, in the formats: mean (standard deviation, SD), median (interquartile range, IQR) or median (range, R). If data were presented in the format from which means and SDs were not extractable, these measures were tried to request from the corresponding author of the publication. If data were presented in the format median(R), our simulations show that the formula “R/4” is the best estimator of the standard deviation (variance) for moderately sized samples (15<n≤70). For large samples (n > 70), the formula “R/6” gives the best estimator for the standard deviation [12]. If data were presented in the format median (IQR), then the formula “IQR/1.35” was used to calculate the standard deviation [13].

### Quality score evaluation

Three authors independently assessed the qualities of included studies using the Newcastle-Ottawa scale (NOS) [14]. The NOS ranges between zero and nine stars. A quality score was calculated on the basis of 3 major components of case–control studies: selection of study groups (0–4 stars), comparability of study groups (0–2 stars), and ascertainment of the exposure of interest (0–3 stars). Studies with a score of seven stars or greater were considered to be of high quality. Disagreement was settled as described above. (Table S2)

### Statistical Analyses

The standardized mean difference (SMD) and 95% confidence interval (95%CI) were calculated for each outcome using a random effect model (the DerSimonian and Laird method). Random effect models were preferable if significant heterogeneity was expected because they assumed and accounted for variable underlying effects in estimates of uncertainty, including both within-study and between-study variance. A Q statistic was calculated using a chi-squared test to quantify the heterogeneity among combined results. A significant Q statistic indicates diversity in the characteristics of the combined trials. Inconsistency was calculated using an I^2^ index to determine the impact of heterogeneity.

To identify potential sources of heterogeneity, planned subgroup analyses were conducted in studies where control subjects were matched for age and gender, in studies excluding subjects with inflammatory comorbidity, and in studies using comparable assay methodology. Planned study level meta-regression analyses were conducted comparing SMDs to pre-operative peripheral inflammatory markers, age, and gender. Sensitivity analysis was used to investigate the influence of each individual study on the overall meta-analysis summary estimate.

The risk of publication bias was assessed using funnel plots and Egger’s tests. Funnel plots were used to plot the effect size of the component studies against the sample size, and Egger’s tests were used to detect skewness in the funnel plots, and by extension, publication bias in the data. The risk of selective reporting bias was examined within the studies. When the difference between groups was not significant, Altman’s method for describing confidence intervals was used. Meta-regression analysis (using metareg) was used to test whether effect sizes were influenced by specific study design features. Analyses were performed using STATA, version 12.0 (Stata Corporation, College Station, Texas, 2012)

## Results

### Literature Search Findings

**Figure 1:**
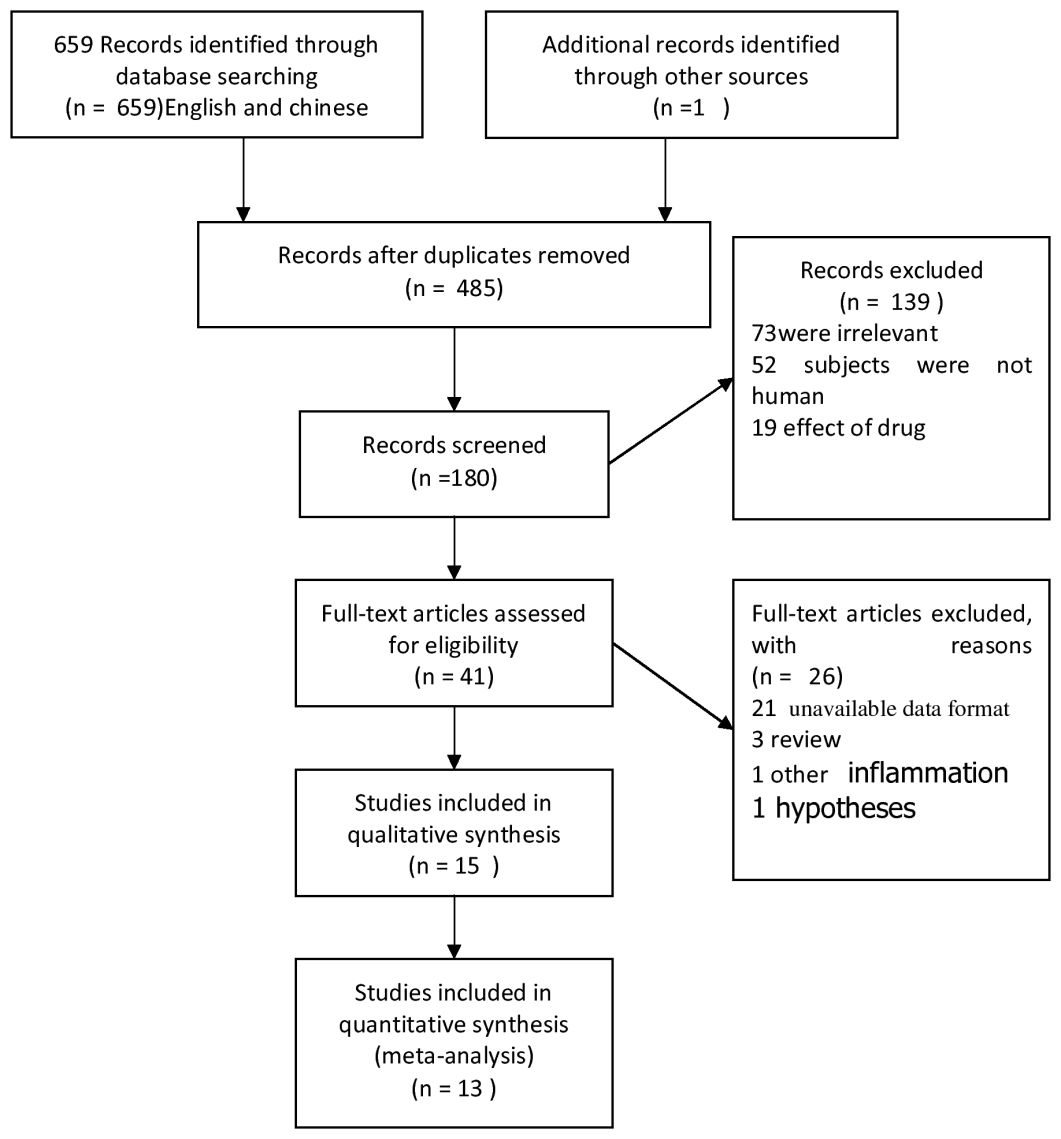
Flow diagram for article selection.

We included 13 eligible studies [2,8–11,15–22] in our meta-analysis (Table S1). In this meta-analysis, we included 7 studies examining S-100β, 3 studies of IL-1β, 5 studies of IL-6, 4 studies of TNF-α, and 6 studies of NSE. Figure 1 shows the study selection procedure.

### Cytokine Concentrations

**Figure 2:**
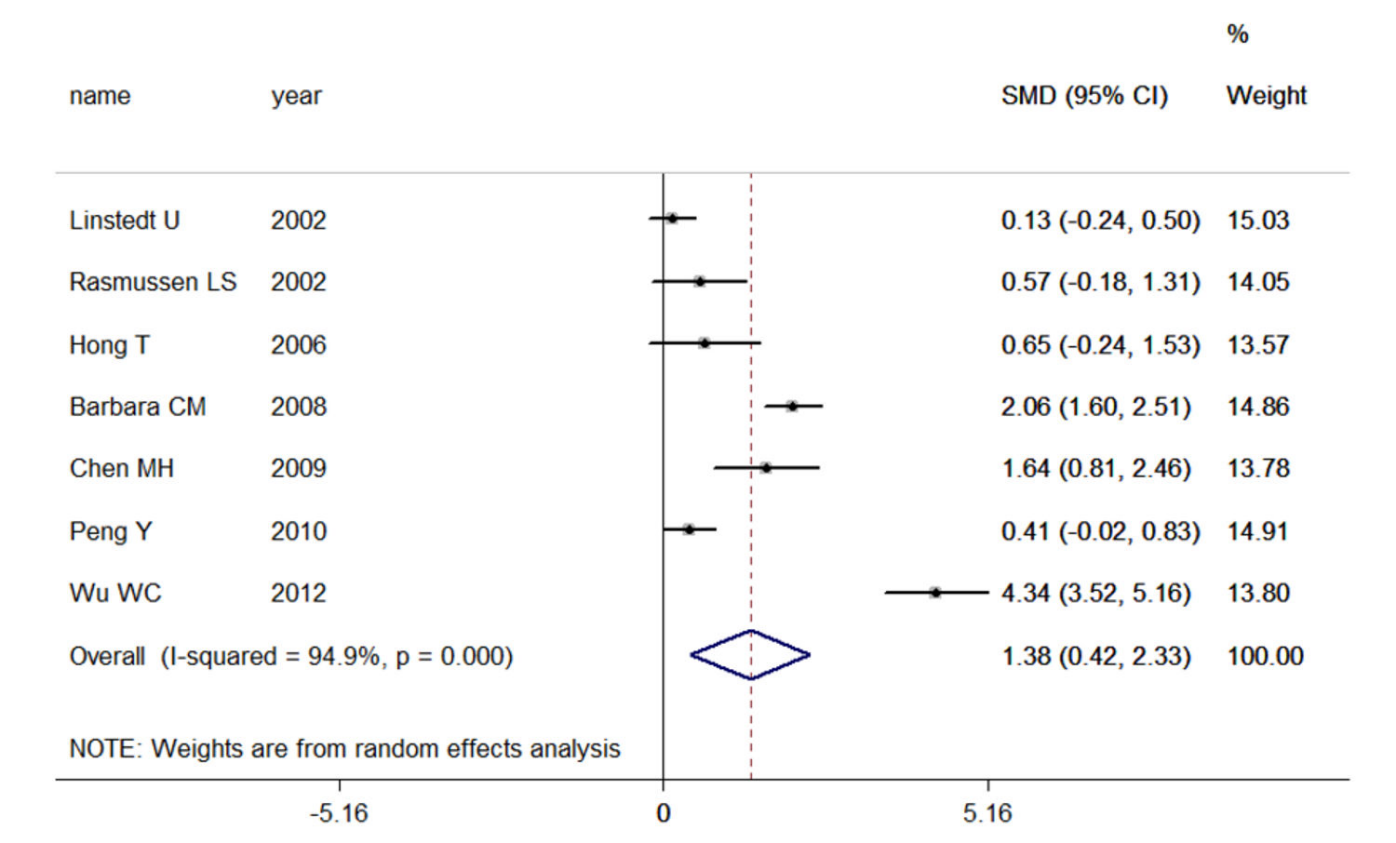
Studies of peripheral blood S-100β. Forest plot for S-100β displaying the results of the random effects meta-analysis for the association between peripheral blood S-100β and postoperative cognitive dysfunction (POCD). The standardized mean difference is presented on the horizontal axis; positive values denote higher serum levels in POCD patients; negative values denote higher serum levels in control subjects. The dashed line is at the overall standardized mean difference. CI, confidence interval; SMD, standardized mean difference.

**Figure 3:**
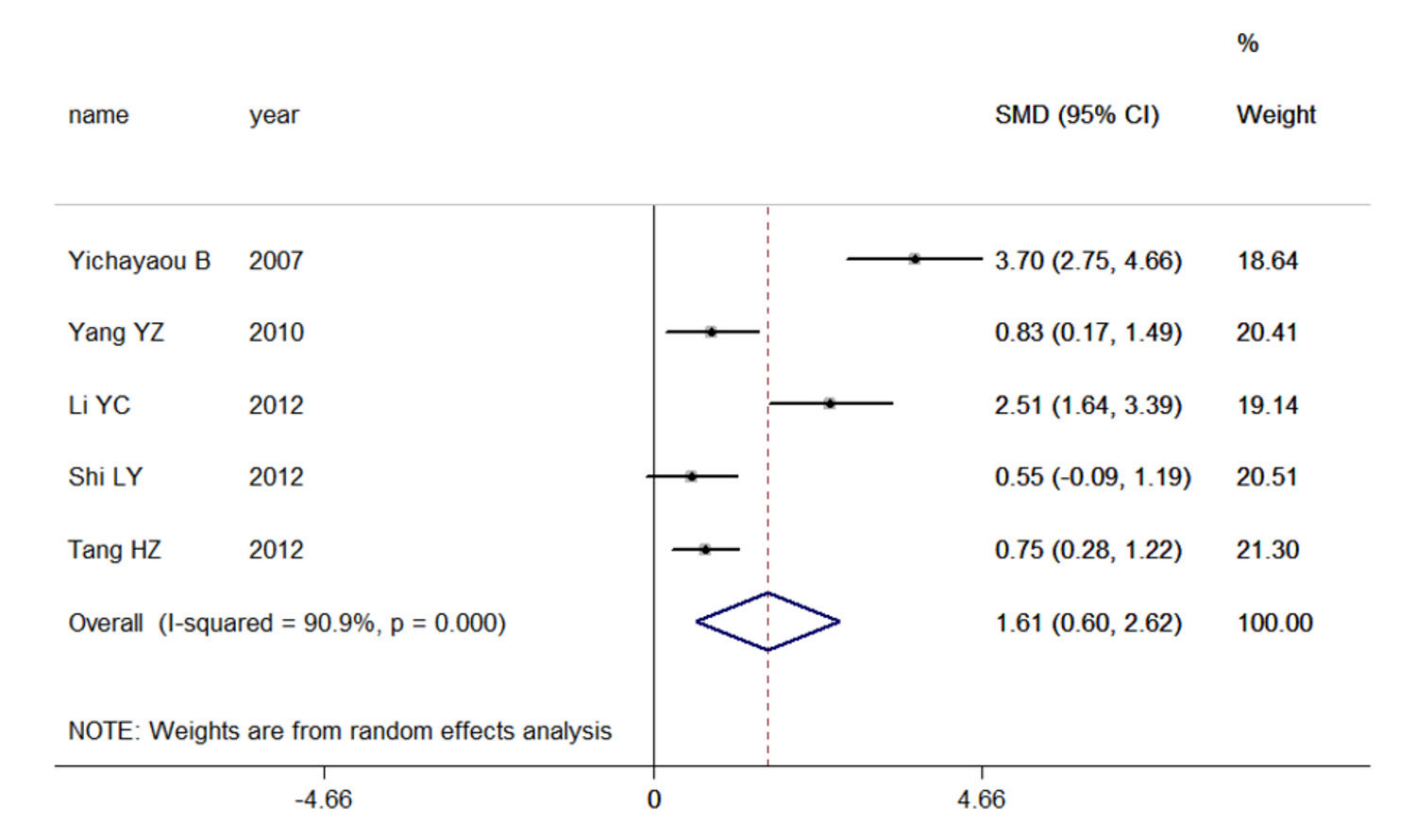
Studies of peripheral blood IL-6. Forest plot for IL-6 displaying the results of the random-effects meta-analysis for the association between peripheral blood IL-6 concentrations and POCD. The standardized mean difference is presented on the horizontal axis; positive values denote higher serum levels in patients; negative values denote higher serum levels in control subjects. The dashed line is at the overall weighted mean difference. CI, confidence interval; SMD, standardized mean difference.

Significantly higher peripheral blood inflammatory marker concentrations (SMD[95%CI]) were detected in POCD subjects compared with non-POCD subjects when examining S-100β(1.377 [0.423 , 2.331], p-value < 0.001 N[POCD/non-POCD] = 178/391,7 studies, Figure 2), and IL-6(1.614 [0.603,2.624], p-value < 0.001, N[POCD/non-POCD ]= 91/99 ,5 studies, Figure 3), but not when examining NSE, IL-1β, or TNF-α. (Table 1)

**Table 1:** Summary of comparative outcomes for each measured peripheral inflammatory marker.

			Main Effect				Heterogeneity			
Cytokine	Studies	N (P/NP)	SMD	95% CI	Z	p	Chi^2^	df	p	I^2^
S-100β	7	178/391	1.377	0.423, 2.331	2.83	0.005	117.35	6	0.000	94.9%
IL-6	5	91/99	1.614	0.603,2.624	3.13	0.002	43.78	4	0.000	90.9%
NSE	6	156/347	0.377	-0.042,0.796	1.76	0.078	20.84	5	0.001	76.0%
IL-1β	3	47/70	0.612	-0.439,1.662	1.14	0.254	14.19	2	0.001	85.9%
TNF-α	4	86/105	0.374	-0.341,1.089	1.03	0.305	16.31	3	0.001	81.6%

S-100β, S-100β protein; IL-6, interleukin-6; NSE, neuron specific enolase; IL-1β, interleukin-1β; TNF-α, tumor necrosis factor-α;N(P/NP), number of patients with POCD/number of patients without POCD;SMD, standardized mean difference (SMD);95% CI, 95% confidence interval

### Investigation of Heterogeneity

All inflammatory marker concentrations were compared in pictograms per milliliter or milligrams per milliliter; however, significant heterogeneity was found in all comparisons. For NSE, an I^2^ index of 76% indicated moderate heterogeneity, but we observed no significance relationship between elevated plasma NSE concentrations and POCD (p-value = 0.078 in 5 studies POCD/non-POCD=135/282). S-100β and IL-6 demonstrate significant relationships with POCD status, but the heterogeneity is high (for S-100β: χ^2^ = 117.35([d.f. = 6] ), p-value = 0.000, I^2^=94.9%, τ^2^=1.5417; for IL-6: χ^2^ = 43.78([d.f. = 4], p-value = 0.000, I^2^ = 90.9%, τ^2^= 1.190) (Table 1).

### Sensitivity analysis

Sensitivity analyses were conducted to ascertain the primary origin of the heterogeneity. For all inflammatory markers, no single study qualitatively changed the SMD, no point estimate of its "omitted" analysis lies outside the confidence interval of the "combined" analysis. Its "omitted" meta-analytic estimate differs in significance relative to the "combined" analysis. Those suggested that the results of this meta-analysis was stable.

### Meta-regression

**Figure 4:**
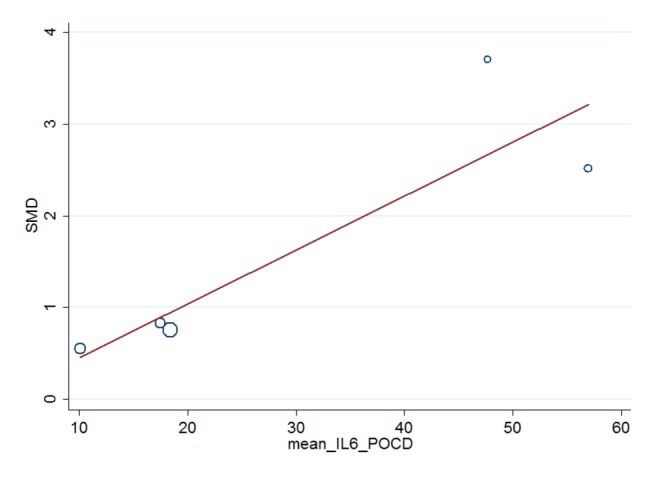
Association of preoperative IL-6 blood concentration in POCD patients with plasma IL-6 SMD using meta-regression. Plot of the preoperative IL-6 peripheral blood concentration vs. the peripheral blood IL-6 SMD in the 5 included studies showing a fitted random effects meta-regression line.( 5 studies, Coef.=0 .0587 ,p-value = 0.038 ) Coef., coefficient.

**Figure 5:**
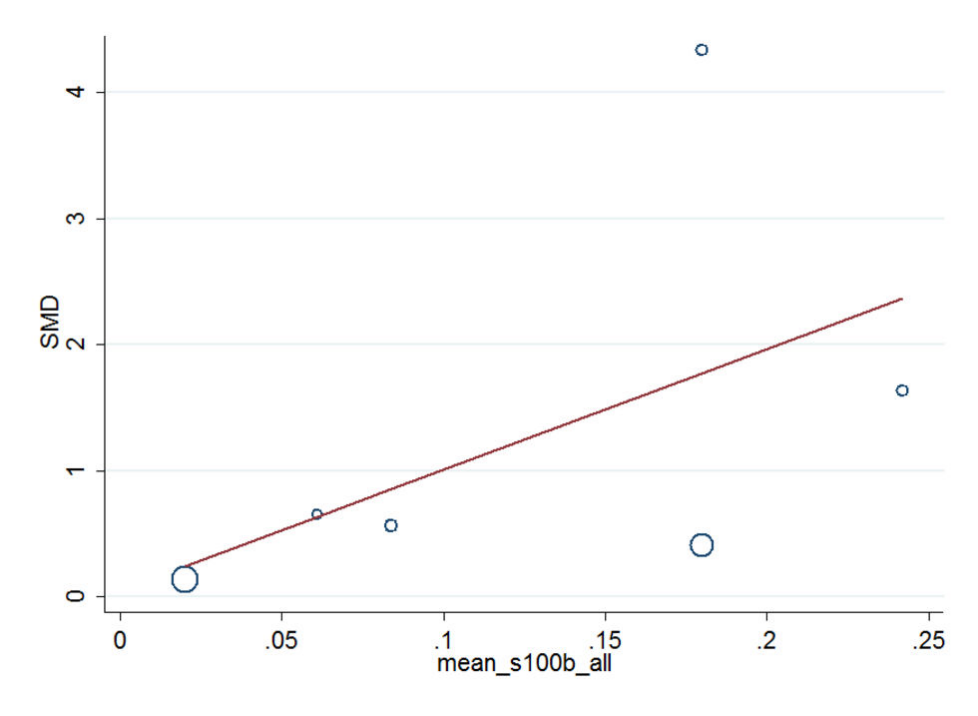
Association of preoperative S-100β blood concentration in all subjects with plasma S-100β SMD using meta-regression. Plot of the preoperative S-100β peripheral blood concentration vs. the peripheral blood S-100β SMD in the 6 included studies, showing a random effects meta-regression line. (6 studies, Coef.= 9.554,p-value = 0.290 ) Coef., coefficient.

Meta-regression analyses were used to assess the associations between the preoperative and postoperative peripheral blood concentrations of the studied inflammatory markers. We conducted this analysis only if the preoperative and postoperative peripheral blood concentrations had at least five measurements from different studies and had either a significant SMD or significant heterogeneity in the absence of a significant SMD. Meta-regression analyses found a significant positive association between the SMD and the preoperative IL-6 peripheral blood concentration in patients with POCD (Coef. = 0.0587, p-value = 0.038, 5 studies, Figure 4 and Table 2). No associations were found between the SMD and the preoperative S-100β peripheral blood concentration (Coef.= 9.554, p-value = 0.290, 6 studies, Figure 5 and Table 2). 

**Table 2:** Meta-regression between the SMDs of postoperative and preoperative S-100β and IL-6 peripheral inflammatory concentrations.

Cytokine	Number of studies	tau2	I^2^ res	Adjusted R^2^	Coef.	Std. Err.	t	P>|t|	[95% CI]
S100β#	6	2.106	94.27%	9.22%	9.554	7.839	1.22	0.290	-12.21,31.32
IL-6##	5	0.2118	61.02%	87.46%	0.0587	0 .0166	3.54	0.038	0.0059 0 .1114

^#^ Preoperative S-100β peripheral blood concentration of all patients

^##^ Preoperative IL-6 peripheral blood concentration of patients with POCD

tau2, estimate of between-study variance by residual maximum likelihood model;

I^2^ res, percentage of residual variation due to heterogeneity;

Adjusted R^2^, Proportion of between-study variance explained;

Coef., coefficient; Std. Err., standard error; 95% CI, 95% confidence interval.

### Publication and Selective Reporting Biases

Significant risk of publication bias was not detected, as demonstrated by funnel plots, and no significant correlations were found between effect size and sample size among studies of peripheral blood S-100β, NSE and inflammatory cytokines, including IL-1β, IL-6 and TNF-α. However, tests for funnel plot asymmetry were only recommended for use when at least 10 studies were included in the meta-analysis. Therefore, Egger’s test was implemented to evaluate asymmetry and publication bias. The results showed no evidence of publication bias: simultaneous reporting of negative results suggested a lower risk of reporting bias; the majority of included studies examined multiple biomarkers, and the majority of studies reported at least one significant comparison also reported at least one non-significant comparison. (Table 3)

**Table 3:** Egger’s test results for publication and selective reporting Bias.

		Egger’s test (bias)			
Cytokine		Coef	95% CI	t	p
S-100β		5.998	-6.389,18.39	1.24	0.268
IL-6		10.19	-1.384 ,21.76	2.80	0.068
NSE		-0.3398	-2.997,2.317	0.74	0.517
IL-1β		23.19	-35.50,81.87	5.02	0.125
TNF-α		-4.995	-40.44,30.45	-0.61	0.606

S-100β, S-100β protein; IL-6, interleukin-6; NSE, neuron specific enolase; IL-1β, interleukin-1β; TNF-α, tumor necrosis factor-α;Coef., coefficient; 95% CI, 95% confidence interval.

## Discussion

A study by van Harten and colleagues showed that an immunological reaction was one of the most important causative factors of cardiac POCD [23]. Buvanendran also found that cardiac and non-cardiac POCD was associated with an inflammatory reaction [24]. In this meta-analysis, we aimed to clarify the association between the inflammatory makers and POCD. Our results show that IL-6 and S-100β are POCD- related pro-inflammatory markers. Although both positive and negative results have been reported in individual studies, this meta-analysis strengthens the clinical evidence that POCD is accompanied by a peripheral inflammatory reaction. Other pro-inflammatory cytokines, such as IL-10 and IL-8, were not involved in this analysis due to the limited number of studies available.

The concentrations of IL-1 and TNF-α did not differ significantly between the subjects with POCD and the control subjects. Following surgery, elderly patients often suffered from POCD, which could persist long after physical recovery. Surgery-induced tissue damage could activate the peripheral innate immune system, resulting the release of inflammatory mediators [25]. 

IL-1β was a pro-inflammatory cytokine that contributed to neuro-inflammation in many central nervous system (CNS) disorders [26] and the development of the inflammatory response within the brain, along with TNF-α and IL-6 [5,27]. In rats, IL-1β messenger RNA (mRNA) was significantly increased at postsurgery days 1 and 3, with an increase in protein expression detected on day 1. There was a significant increase in TNF-α mRNA on day 1 after surgery, although there was no increase in protein expression [28].

It was possible that our findings could not be interpreted as truly negative due to small sample sizes and significant heterogeneity between studies. Additional evidence demonstrated inflammatory activity with POCD, which could be the mechanism through which peripheral pro-inflammatory markers induced POCD. In mouse models, Barrientos and colleagues demonstrated that a single intracisternal administration of interleukin-1 receptor antagonist (IL-1RA) at the time of surgery was sufficient to block both the behavioral deficits and the neuro-inflammatory response. Injecting the same dose of IL-1RA peripherally failed to have a protective effect. These data provided strong support for the specific role of central, but not peripheral, IL-1β in POCD [29]. 

Following surgery under general anesthesia, TNF-α was the first cytokine to be released, and its concentration peaked (p-value < 0.05) 30 min after surgery. Terrando explained that TNF-α was important in cognitive decline. TNF-α was upstream of IL-1 and stimulates its production in the brain. Peripheral blockade of TNF-α could limit the release of IL-1 and prevent neuro-inflammation and cognitive decline in a mouse model of surgery-induced cognitive decline [30]. Belarbi found that the TNF-α synthesis inhibitor DT(3,6’dithiothalidomide) could significantly reverse hippocampus-dependent cognitive deficits induced by chronic neuro-inflammation. These results suggested that TNF-α was a critical mediator of chronic neuro-inflammation-induced neuronal dysfunction and cognitive impairment, and targeting its synthesis could be an effective therapeutic approach for several human neuron degenerative diseases [31].

Although POCD was a common complication of major surgery, its pathogenic mechanisms remained unknown. However, the contribution of several risk factors, including advanced age, duration of anesthesia, a second operation, low education level, postoperative infection, and respiratory complications, have been identified [4]. The apolipoproteinE4( ApoE4) allele was a known risk factor for Alzheimer’s disease [32], and one large study found a strong association between the ApoE4 allele and POCD in elderly patients who were given inhaled anesthetics [33]. However, McDonagh and colleagues were unable to found an association between cognitive decline and ApoE4 genotype, despite a robust sample size. Similarly, they also did not found association between perioperative B-type natriuretic peptide, CRP, D-dimer, matrix metalloproteinase-9, NSE, or S-100β levels and either POCD or the ApoE4 genotype [34].

A clinically relevant concentration of β-amyloid generation and aggregation was a key factor in the pathogenesis of Alzheimer’s disease [35]. These findings suggested that such an accumulation in elderly patients could potentially increase the risk of POCD. Wan et al. suggested that surgery could provoke astrogliosis, β-amyloid accumulation, and τ phosphorylation in elderly patients, which was likely to be associated with the cognitive decline [36].

Lipopolysaccharide (LPS) came from gram-negative bacteria and it was an endotoxin that could cause a strong immune response. When LPS bound the CD14/Toll-like receptor 4 /MD^2^ complex, it could stimulate the inflammatory cells to secrete a variety of cytokines. Some studies have examined the relationship between LPS and POCD [6, 37]. 

Observational studies could not clearly infer a causal relationship. Thus, the possibility that increased concentrations of pro-inflammatory cytokines were a consequence of POCD pathology should also be considered. Cibelli and colleagues explored whether systemic inflammation in response to surgical trauma triggers hippocampal inflammation and subsequent memory impairment in a mouse model of orthopedic surgery. Their studies suggested that inflammation play a critical role in the pathogenesis of POCD, as evidenced by the protection afforded to surgical animals by minocycline which was a nonspecific inhibitor of inflammation [5].

Peripheral pro-inflammatory signals can be actively propagated across the blood brain barrier (BBB) via crosstalk between peripheral and central immune cells [37]. Cytokines produced peripherally could cross the BBB via saturable transporters [38,39] or by passive diffusion through spaces between vascular endothelial cells [40]. Microglia was important in brain inflammation and inflammatory neurodegeneration as the resident innate immune cells of the CNS. Increasingly evidence illustrated that microglia played a crucial role in regulating both pathogenic and subsequent repair processes [41,42]. Guy C. Brown found that activation was accompanied by partial rounding up and mobility of the cells, proliferation, and the expression and release of pro-inflammatory cytokines, including TNF-α, IL-1 β, and IL-6 in the event of brain inflammation. These cytokines activated other microglia and astrocytes [43]. Microglia cells might secrete a variety of inflammatory mediators and neurotoxic factors [44]. Peripheral inflammatory markers might directly or indirectly cause a CNS inflammatory response. When excessive CNS inflammatory reactivity occurred, peripherally originating inflammatory markers could influence cognitive function by interfering with neuronal activity, which affected the function of synaptic connections, or by causing neuron toxicity and neuron degeneration, which resulted in impaired cognitive function [2].

The S-100β protein has been used as a marker of neuronal damage in various diseases [45]. S-100β belongs to the family of calcium-binding proteins. It was expressed primarily by astrocytes and found both intra- and extra-cellularly in brain tissue [46]. S-100β was usually elevated in the blood and CSF following nervous system damage due to a functional disturbance of membrane integrity and/or increased permeability of the BBB. Therefore, the S-100β protein might be a potential marker for POCD.

Li and colleagues found that the serum levels of the pro-inflammatory markers IL-6 and S-100β increased after general anesthesia in total hip-replacement surgery, and these increases may be associated with the occurrence of POCD [2].In our meta-analysis, we provide statistical evidence to support this association. Studies have shown that enhanced S-100β upregulated the cyclooxygenase-2 expression by way of RAGE in microglia [47], and in those with Alzheimer disease cyclooxygenase-2 inhibitor impeded neuro-inflammation and induced amelioration of cognitive function [48]. Rui-Lin Li indicated that RAGE signal transduction pathways mediated S-100β-induced neuro-inflammation, which might play a critical role in driving the pathogenesis of cognitive decline after surgery. And it could also activate the nuclear factor κB (NF-kB) signal pathways and gave rise to upregulation of pro-inflammatory mediators, such as IL-1β [49]. It is difficult to distinguish cause and effect in the relationship between inflammation and cerebral tissue damage. In the rat brain, both experimental subarachnoid hemorrhage [50] and global forebrain ischemia [51] induced upregulation of inflammatory gene pathways, as represented by the transcription factor NF-κB. Moreover, focal cerebral ischemia caused not only local inflammation but also widespread cerebral inflammation [52]. 

POCD is a growing and largely underestimated problem without a defined etiology. Globally, experts were focused on discovering risk factors for the occurrence of POCD and developing precautions to reduce its incidence rate [53–55], but results have thus far been inconsistent. Meta-regression analyses utilizing the subgroup of studies reporting preoperative mean IL-6 levels suggested that the postoperative level of some inflammatory markers might have contribution to the occurrence of POCD. In Cibelli model, IL-6 plasma levels and transcription in the hippocampus appeared to increase after surgery. IL-6 had facilitating effects on IL-1β in mediating inflammation and causing hippocampal-dependent memory impairment [5]. Hudetz indicated that elevated postoperative IL-6 and CRP concentrations were associated with the subsequent development of short-term and medium-term impairment of cognitive functions after coronary artery surgery [56]. The relationship between inflammation and cognitive deficit was studied in a healthy young population after infusion of IL-6 to simulate the physiological acute-phase response, and the results indicated an explicit decrease in self-reported concentration and cognitive abilities [57]. The meta-regression analyses also expressed the same tendency. Assessment of hippocampal expression of the pro-inflammatory cytokines IL-6 confirmed that aged mice showed compared to adults embraced higher basal expression of these inflammatory genes in agreement with prior reports that showed aging primes microglial cells [58].

 As the global aging progresses, the search for new preventative measures and treatment strategies to maintain higher brain functions throughout life is of major economic and medical importance [59]. In recent years, numerous studies have suggested that multiple strategies, including aerobic exercise, art therapy, and caloric restriction could enhance cognitive fitness [60–62]. It would improved perioperative brain functional recovery that searching for biomarkers of brain functional reserve and the methods which could enhance it. IL-6 might be a biomarker that could guide the prevention and treatment of POCD. There was growing interest in inflammation-targeted therapeutics. 

The present meta-analysis examining the role of peripheral inflammatory marks in POCD has several methodological limits. The literature search was limited to unstimulated cytokines (IL-1β, IL-6, TNF-α), NSE, and S-100β; however, other inflammatory markers (e.g. IL-10, high mobility group box 1(HMGB-1), NF-κB) have also been informative [63,64]. This meta-analysis was also limited by the substantial inconsistency in most comparisons, necessitating the use of random effects models that produced wider confidence intervals for most inflammatory markers(e.g. S-100β, IL-6, IL-1β, and TNF-α) .Most studies reported large standard deviations, suggesting substantial unexplained inter-individual variation in inflammatory marker concentrations. Some potential sources of heterogeneity, including assay methodology, age, gender, and medical comorbidity, were investigated. 

The present study was also limited by the use of a categorical diagnosis of POCD; thus, POCD severity effects could not be systematically investigated. Meta-regression analyses in subgroups of studies reporting preoperative mean IL-6 and S-100β protein levels suggested that the preoperative level of some inflammatory markers might have contributed to the heterogeneity.

Furthermore, in cross-sectional studies, continuous and stochastic elevations could not be distinguished, and differences in the secretion patterns and half-lives of different cytokines might have a considerable impact on their measured concentrations. Most inflammatory markers immediately reached the peak postoperative value [30], but the peak serum concentration was not always the most valuable measure in the prediction of outcome [10]. One study found that the early and pronounced release of inflammatory markers was clinically unimportant but that the increase after 4-48h was significantly higher in patients with POCD than in patients without POCD [9].

Differences in enzyme linked immunosorbent assay (ELISA) techniques were investigated as a potential source of heterogeneity; however, many other assay parameters and methodological differences in the collection and handling of the samples that could not be systematically addressed might have contributed to heterogeneity.

Funnel plots and Egger's tests did not suggest the presence of publication bias. However, many observational studies were not registered with clinical trials data-bases, so the scope of the unpublished literature could not be ascertained and effects of bias at the study and outcome levels could not be ruled out. In addition, this article was mainly based on studies published in the English and Chinese language, and bias might have exist in the literature published in other languages. 

In conclusion, our meta-analysis provides evidence that POCD is indeed correlated with the concentrations of IL-6 and S-100β. Furthermore, the meta-regression shows that IL-6 might serve an indicator to guide the prevention and treatment of POCD. Because POCD is a common postoperative complication and is related multiple factors, further investigation of the inflammatory response regulatory pathways, and the specific contributions of IL-6 and gene polymorphisms is necessary and meaningful.

## Supporting Information

Checklist S1 **Reporting items for the meta-analyses.**
(DOC)

Table S1 **Characteristics of the association studies that were included in the systematic review and that examined the S-100β and peripheral inflammatory markers.**
(DOC)

Table S2 **Newcastle-Ottawa quality assessment scale for evaluation to the quality of each study.**
(DOC)
