# Supplementary material for: Role of Peripheral Inflammatory Markers in Postoperative Cognitive Dysfunction (POCD): A Meta-Analysis
Source: PLoS One. 2013 Nov 13;8(11):e79624. doi: 10.1371/journal.pone.0079624 (PMC3827367; doi:10.1371/journal.pone.0079624)
Supplement: Table S1 — Characteristics of the association studies that were included in the systematic review and that examined the S-100β and peripheral inflammatory markers. (DOC) [file pone.0079624.s002.doc]

TableS1.Characteristics of the association studies that were included in the systematic review and that examined the S-100β and peripheral inflammatory markers.

|  |
| --- |

|  | ID | First author | Year | Number of  POCD/Non-POCD^a^ | Gender(F/M)ratio in POCD/Non-POCD | Age(years) of  POCD/Non-POCD | ASA | Type of anesthesia | Type of surgery | Peripheral inflammatory factors | Diagnostic  criteria or method |
| --- | --- | --- | --- | --- | --- | --- | --- | --- | --- | --- | --- |
|  | 1 | Wu  WC[16] | 2012 | 21/65 | Total(F/M)  40/46 | Total(Mean, SD)71.39 ±  5.72 | NA | Spinal block  anesthesia | Non-cardiac surgery | NSE, S-100β | SECF* |
|  | 2 | Tian HZ[14] | 2012 | 39/34 | POCD(F/M): 15/24  Non-POCD(F/M):  13/21 | POCD/non-POCD(mean, SD)51.46± 17.93/45.29 ± 15.50 | I-III | NA | Non-cardiac surgery | TNF-α, IL-6, hs-CRP | MMSE** |
|  | 3 | Hong T[20] | 2006 | 7/19 | Total(F/M)：  12/14 | Total(Mean, SD)76±5 | II-III | Intravenous-inhalation anesthesia | Non-cardiac surgery | S-100ββ | MMSE  AMT*** |
|  | 4 | Shi LY[21] | 2012 | 17/23 | POCD(F/M):11/6  Non-POCD(F/M): 8/15 | POCD/non-POCD(Mean, SD)61.5±12.3/53.9±16 | I-II | General anesthesia | Non-cardiac surgery | IL-6,IL8,IL1ra,TNFRII,IL-1β,TNF-α | MMSE  BCAI**** |
|  | 5 | Chen MH[19] | 2009 | 9/31 | Total(F/M):17/23 | Total(Mean, SD)65.2±10. | II | IIA**#  Spinal | Non-cardiac surgery | S-100β | MMSE |
|  |  |  |  |  |  | 2 |  | block anesthesia |  |  |  |
|  | 6 | Xu YH18] | 2010 | 16/24 | POCD(F/M):12/4  Non-POCD  (F/M):15/9 | Total(Mean, SD)71.2±5.9 | I-II | IIA | Non-cardiac surgery | NSE | MMSE |
|  | 7 | PengY  [17] | 2010 | 28/92 | POCD  (F/M):16/12  Non-POCD  (F/M):52/40 | POCD/non-POCD(Mean, SD)68.2±5.8/  67.8±6.1 | I-II | General anesthesia | Non-cardiac surgery | S-100β,NSE | DSM-IV# |
|  | 8 | Yang  ZY[15] | 2010 | 18/21 | NA | Total≥70 | NA | NA | Non-cardiac surgery | IL-1β,IL-6,TNF-α | MMSE |
|  | 9 | BelooseSy Y[11] | 2007 | 12/29 | Total(F/M):  29/12 | Total(Mean, SD)81.8 ±7.7 | I+II:29  III+IV:12 | NA | Non-cardiac surgery | CRP,IL-1β,IL-6,IL-8,TNF-α,IL-10,IL-1ra | DSM-IV  MMSE  CAM## |
|  | 10 | Rasmussn LS[10] | 2000 | 8/57 | Total(F/M):  16/49 | Total(Median, IQR)  68( 65-72 ) | NA | NA | Non-cardiac surgery | S-100β,NSE | DSM-III  MMSE |
|  | 11 | VanMunser BC[8] | 2009 | 62/58 | POCD  (F/M):46/16  Non-POCD  (F/M):35/23 | POCD/non-POCD(Mean, SD)84.8±6.9 /82.9± 7.0 | NA | Spinal block anesthesia | Non-cardiac surgery | IL-6,IL-8,IL-12,S-100β,NSE | CAM  DOS###  DRS-R-98#### |
|  | 12 | Li YC[2] | 2012 | 17/20 | POCD  (F/M):10/7  Non-POCD  (F/M):12/8 | Total≥60 | ＞III | General anesthesia | Non-cardiac surgery | S-100β,IL-1β,IL-6,TNF-α,CRP | DSS*#  Concentration endurance  Test d2 |
|  | 13 | LinstedtU[9] | 2002 | 48/69 | NA | Total(Median,R)64(18–85) | NA | NA | Non-cardiac surgery | S-100β,NSE | DSS |

Year: published year

Number POCD/non-POCD: the number of patients with POCD versus without POCD

Gender(F/M) ratio in POCD/Non-POCD: the ratio of female to male in patients with POCD versus without POCD;

Total(F/M): the ratio of female to male regardless of the diagnosis of POCD;

POCD(F/M):the ratio of female to male in patients with POCD;

Non-POCD(F/M): the ratio of female to male in patients without POCD.

Age(years)POCD/Non-POCD: the age of patients with POCD versus without POCD in year;

Total(mean, SD): the age of patients regardless of the diagnosis of POCD in the format of mean(standard deviation, SD);

POCD/non-POCD(mean, SD): the age of patients with POCD versus without POCD in the format of mean(standard deviation, SD);

Total(Median, IQR): the age of patients regardless of the diagnosis of POCD in the format of median(interquartile range, IQR );

Total(median, R): the age of patients regardless of the diagnosis of POCD in the format of median(range, R);

Total: the status of patients regardless of the diagnosis of POCD without more detailed description.

ASA: American Society of Anesthesiologists Classification of Anesthesia Risk

NA: Not Available

CRP, C-reactive protein;

hs-CRP, high-sensitivity C-reactive protein;

IL-1β, interleukin-1β;

IL-1ra, interleukin-1 receptor antagonist

IL-6, interleukin-6;

IL-8, interleukin-8;

IL-10, interleukin-10;

IL-12, interleukin-12;

NSE, neuron specific enolase;

S-100β,S-100βprotein;

TNF-α,tumor necrosis factor-α.

* SECF: Scale of Elderly Cognitive Function

** MMSE: Mini-mental State Examination

***AMT: Abbreviated Mental Test

**** BCAI: Battery of Cognitive Assessment Instruments for Elderly

# DSM: Diagnostic and Statistical Manual of Mental Disorders

## CAM: Confusion Assessment Method

### DOS: Observation Screening Scale

#### DRS-R-98: Delirium Rating Scale-Revised-98

*# DSS: Digit-Symbol-Substitution Test

**# IIA: Intravenous inhalation anesthesia
