# Supplementary material for: Role of Peripheral Inflammatory Markers in Postoperative Cognitive Dysfunction (POCD): A Meta-Analysis
Source: PLoS One. 2013 Nov 13;8(11):e79624. doi: 10.1371/journal.pone.0079624 (PMC3827367; doi:10.1371/journal.pone.0079624)
Supplement: Table S2 — Newcastle-Ottawa quality assessment scale for evaluation to the quality of each study. (DOC) [file pone.0079624.s003.doc]

**Table S2. Newcastle-Ottawa quality assessment scale for evaluation to the quality of each study**

|  |  | Selection | | | | Comparability | | Exposure | | |
| --- | --- | --- | --- | --- | --- | --- | --- | --- | --- | --- |
| ID | First author | #1 | #2 | #3 | #4 | #5 | #6 | #7 | #8 | #9 |
| 1 | WuWC[16] |  |  |  |  |  |  |  |  |  |
| 2 | Tian HZ[14] |  |  |  |  |  |  |  |  |  |
| 3 | Hong T[20] |  |  |  |  |  |  |  |  |  |
| 4 | Shi LY[21] |  |  |  |  |  |  |  |  |  |
| 5 | Chen MH[19] |  |  |  |  |  |  |  |  |  |
| 6 | Xu YH[18] |  |  |  |  |  |  |  |  |  |
| 7 | PengY[17] |  |  |  |  |  |  |  |  |  |
| 8 | YangZY[15] |  |  |  |  |  |  |  |  |  |
| 9 | BelooseSyY[11] |  |  |  |  |  |  |  |  |  |
| 10 | RasmussnLS[10] |  |  |  |  |  |  |  |  |  |
| 11 | VanMunserBC[8] |  |  |  |  |  |  |  |  |  |
| 12 | Li YC[2] |  |  |  |  |  |  |  |  |  |
| 13 | LinstedtU[9] |  |  |  |  |  |  |  |  |  |

#1.Diagnosis of POCD

#2.Representativeness of patients with POCD

#3.Selection of the Comparative patients without non-POCD

#4.Definition of Controls

#5.Study controls for age

#6.Study controls for Gender/ASA/Type of surgery/Type of anesthesia/Education years/Time of surgery

#7.Ascertainment of POCD station

#8.Same method of ascertainment for POCD station

#9. Lost to follow up acceptable

Note: Each study was assessed based on three broad perspectives: selection, comparability, and exposure with a score ranging from 0 to 9. A score of 7 or greater indicated that one study was of high quality.

1. Selection: According to the definition of Postoperative cognitive dysfunction (POCD), the cognitive dysfunction was thought to be associated with cardiac or non-cardiac surgery, and elderly individuals tend to appear an alteration in orientation, memory, thinking, attention, insight or other aspects of central nervous function. As for controls, for ethic reasons, fake surgery is unavailable; therefore hospitalized elderly individuals who accept surgery are selected. That is to say, both of case and control derived from a hospitalized population. All eligible cases including patients with or without POCD over a defined period of time, and in a defined hospital. On the other hand, the peripheral inflammatory markers may be measured repeatedly. To avoid confusions of complications and past history, most of cases are diagnosed POCD or even cognitive dysfunction firstly, and controls are stated no history of cognitive defects.

2. Comparability: Either cases and controls was matched in the design. Age as the most important confounder was adjusted for in the analysis. Statements of no differences between groups or that differences were not statistically significant are not sufficient for establishing comparability. There are other controlled factors such as Gender/ASA/Type of surgery/Type of anesthesia in some articles.

3. Exposure: Ascertainment of exposure was tested by structured interview where blind to case/control status in the same method. Non-Response rate was basically same for both groups. Death and/or life-threatening sever complication after surgery were main reasons for patients’ withdrawal from experiment, which may contribute to bias.
